# Abnormal characteristic static and dynamic functional network connectivity in idiopathic normal pressure hydrocephalus

**DOI:** 10.1111/cns.14178

**Published:** 2023-03-22

**Authors:** Wenjun Huang, Xuhao Fang, Shihong Li, Renling Mao, Chuntao Ye, Wei Liu, Yao Deng, Guangwu Lin

**Affiliations:** ^1^ Department of Radiology Huadong Hospital Affiliated to Fudan University Shanghai China; ^2^ Department of Neurosurgery Huadong Hospital Affiliated to Fudan University Shanghai China

**Keywords:** dynamic, functional network connectivity, idiopathic normal pressure hydrocephalus, independent component analysis, resting‐state functional magnetic resonance imaging, static

## Abstract

**Aims:**

Idiopathic Normal pressure hydrocephalus (iNPH) is a neurodegenerative disease characterized by gait disturbance, dementia, and urinary dysfunction. The neural network mechanisms underlying this phenomenon is currently unknown.

**Methods:**

To investigate the resting‐state functional connectivity (rs‐FC) abnormalities of iNPH‐related brain connectivity from static and dynamic perspectives and the correlation of these abnormalities with clinical symptoms before and 3‐month after shunt. We investigated both static and dynamic functional network connectivity (sFNC and dFNC, respectively) in 33 iNPH patients and 23 healthy controls (HCs).

**Results:**

The sFNC and dFNC of networks were generally decreased in iNPH patients. The reduction in sFNC within the default mode network (DMN) and between the somatomotor network (SMN) and visual network (VN) were related to symptoms. The temporal properties of dFNC and its temporal variability in state‐4 were sensitive to the identification of iNPH and were correlated with symptoms. The temporal variability in the dorsal attention network (DAN) increased, and the average instantaneous FC was altered among networks in iNPH. These features were partially associated with clinical symptoms.

**Conclusion:**

The dFNC may be a more sensitive biomarker for altered network function in iNPH, providing us with extra information on the mechanisms of iNPH.

## Graphical abstract

The current study is the first study in iNPH cohort to combine a dynamic analysis framework with classical sFNC to investigate changes in brain connectivity from both static and dynamic perspectives, providing extra information on the underlying mechanisms of iNPH.

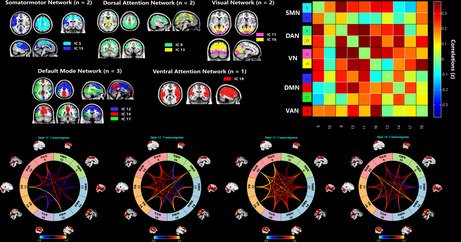

## 1 INTRODUCTION

Idiopathic normal pressure hydrocephalus (iNPH), a neurodegenerative disease that presents as by gait disturbance, cognitive impairment, and urinary incontinence of unknown origin, is characterized by ventricular enlargement despite cerebrospinal fluid (CSF) pressure being within the normal range.^1^, ^2^ The definite diagnosis depends on symptom reversal after CSF tap test (TT) and shunt surgery.^2^ The degree of symptom reversal after shunting varies according to the condition of the patient.^3^, ^4^, ^5^ Neither the pathophysiological mechanisms nor the neural networks underlying this phenomenon have been elucidated to date.

Resting‐state functional MRI (rs‐fMRI) could detect the highly localized alterations of brain network activation and connectivity at rest to identify the underlying neural sources.^6^, ^7^, ^8^ Previous large‐scale network analysis studies using independent component analysis (ICA) and region of interest (ROI)‐based approaches in iNPH revealed the alterations in intra‐ and internetwork connectivity, which might be related to the severity of symptoms and the efficacy of shunt surgery.^6^, ^7^, ^9^ However, the temporal resolution of rs‐fMRI is longer than necessary to study the dynamics of brain function, and most previous rs‐fMRI studies were based on the assumption of spatial and temporal stationarity in the resting‐state, which may limit their results.^10^ Recently, studies involving psychiatric and neurodegenerative diseases indicated that dynamic functional network connectivity (dFNC) might contribute to capturing transient signs of damage in brain tissue that cannot be observed from static analysis and to providing important information on the underlying nature.^11^, ^12^ Bommarito G et al. provided what is, to our knowledge, the first in‐depth description of iNPH through resting‐state dynamics. Alterations in interactions between large‐scale networks explained iNPH symptoms, and the normalization of these alterations after CSF‐TT may contribute to prognosis prediction and differential diagnosis.^9^

A framework for dFNC and static FNC (sFNC) based on group ICA has been proposed to compare the whole‐brain FNC features between Alzheimer's disease (AD) and subcortical ischemic vascular disease (SIVD).^11^ DFNC focuses on connectivity between networks^13^, ^14^ and has been widely employed in identifying dFNC biomarkers for different psychiatric and neurodegenerative diseases.^13^, ^15^, ^16^

The primary aim of the current work was to investigate the resting‐state functional connectivity (rs‐FC) abnormalities of iNPH brain connectivity and the correlations of these abnormalities with clinical symptoms from static and dynamic perspectives. We hypothesized the presence of changes in sFNC and dFNC between iNPH patients and healthy controls (HCs). We also assumed that the altered FNC features would be associated with the clinical symptoms of iNPH and that dFNC could provide additional biomarkers for supplying sFNC.

## 2 MATERIALS AND METHODS

### 2.1 Participants

This study was performed between February 2021 and March 2022, which approved by the Ethics Committee of Huadong Hospital affiliated with Fudan University (approval number: 2017K027). A total of 80 participants, diagnosed iNPH patients (*n* = 47) and healthy persons of a similar age (*n* = 23) were included in this retrospective cross‐sectional study. INPH patients were recruited at the Department of Neurosurgery of Huadong Hospital affiliated to Fudan University to undergo shunt surgery. All iNPH patients fulfilled the criteria for possible, probable, and definite iNPH based on the third edition of the guidelines for management of iNPH.^2^ See Appendix S1 for details. Finally, 33 patients with diagnosed iNPH (26 male, 7 female; 74.03 ± 8.94) were included in this study. Twenty‐three elderly healthy control subjects (14 male, 9 female; 75.57 ± 6.66) were included for comparison. The two groups had no significant demographic differences in age (*p* > 0.05).

Clinical symptoms assessments can be found in Appendix S1.

### 2.2 MR imaging acquisition and functional data preprocess

MR imaging data were obtained with a 3‐T whole‐body MRI scanner (MAGNETOM Prisma, Siemens Healthcare, Erlangen, Germany). The scanning parameters of functional images and structure images can be found in Appendix S1. Imaging data were preprocessed using a standard pipeline (Appendix S1).

### 2.3 Group independent component analysis

After data preprocessing, resting‐state data of all participants were analyzed using spatial ICA as implemented in the Group ICA Of fMRI Toolbox (GIFT v4.0b, http://mialab.mrn.org/software/gift) to create the intrinsic connectivity networks used in our analysis. Detailed process displayed in Appendix S1. After that, we sorted 10 meaningful ICs into seven functional networks, based on the spatial correlation values between ICs and the template.^17^

### 2.4 Statistic functional network connectivity (sFNC) analysis

We calculated sFNC using the Pearson correlation coefficients between the time courses of ICs, then obtained an sFNC matrix with the dimension of 10 × 10 (10 is the number of ICs) and an sFNC‐domain matrix with the dimension of 5 × 5 (5 is the number of large‐scale networks which contain all the ICs) for each participant. The coefficients were extracted when statistically significant group differences were observed in sFNC. After that, correlation analysis was used to compute the correlation between coefficients and clinical scores.

### 2.5 Dynamic functional network connectivity (dFNC) analysis

The common two ways to investigate dFNC are the sliding window approach and k‐means clustering. The sliding‐window approach was used to explore time‐varying changes of FC within 10 ICs during functional MRI scans. Resting‐state time series data were segmented into a 30TR window with a Gaussian of *σ* = 3 TRs,^18^ which resulting in 170 consecutive windows across the entire scan. As covariance estimation using shorter time series can be noisy, the regularized inverse covariance matrix was used.^19^ To promote sparsity in estimation, a penalty on the L1‐norm (Manhattan distance) was imposed in the graphic LASSO framework with 100 repetitions.^20^ After computing dFNC, all the FC matrices were transformed to z‐scores using Fisher's z‐transformation to stabilize variance and were residualized with nuisance variables (such as age and gender) prior to further analysis.

### 2.6 Functional connectivity state analysis

We applied *k*‐means clustering methods on 170 window FC matrices for all subjects to estimate reoccurring FC patterns (states), which could be characterized by both frequency and structure based on previous methods.^10^ *K* = 4 was determined using the elbow criterion.^10^ We repeated the clustering algorithm 500 times to increase the chance of escaping the local minima.^10^ All‐time windows of all subjects were clustered into four states based on the similarity with the cluster centroid, then we investigated the average instantaneous functional connectivity and temporal properties of dFNC states by computing the fractional time (i.e., the number of total windows belonging to one state) and mean dwell time (i.e., the number of consecutive windows belonging to one state before changing to the other state) in each state as well as the transition number (i.e., the number of transitioning between states and represents the reliability of each state) between different states for each subject.

Group differences in mean dwell time, fractional windows, and transition number, between age‐matched iNPH patients and HCs were examined using two‐sample *t*‐test [*p* < 0.05, false discovery rate (FDR) correction].

### 2.7 Temporal variability of independent components

We investigated the between‐group differences in the temporal variability of each IC. Specifically, we first measured the temporal stability of FNC in IC, that is, *k*, by the average Pearson correlation coefficient between the *k*‐th row of every two correlation matrices of each participant, and then, the temporal variability *V*_*k*_ of IC k can be described by one minus the temporal stability.^21^, ^22^

$$V_{k}=1-\frac{\Sigma_{i\neq j}\rho_{F_{i,k},F_{j,k}}}{n\times \left(n-1\right)}$$

In which *n* = 170 is the total number of windows and $\rho_{F_{j,k},F_{j,k}}$ is the Pearson correlation coefficient between the FNC of IC *k* in the correlation matrices derived from the *i*‐th and the *j*‐th windows (*i*, *j* = 1, 2, …, *n*; *i* ≠ *j*; *k* = 10).

### 2.8 Data analysis

Demographic and clinical data, evaluated with the Kolmogorov–Smirnov test for normality, were reported as means and standard deviations or medians and interquartile ranges for continuous variables, or as percentages for categorical variables. All functional data (including sFNC, dFNC, mean dwell time, fractional windows, transition number and average instantaneous functional connectivity) followed normal distribution because Fisher's z‐transformation had been performed on all functional data.

The two‐sample *t*‐test was used to analyze the normalized data, otherwise the Mann–Whitney non‐parametric tests for non‐normalized data. The paired‐sample *t*‐test was used to analyze the clinical symptoms change in the iNPH patients before and 3 months after shunting, and the change was presented as percentage (ratio of difference to preoperative score). The correlation analysis *p* < 0.05 was considered statistically significant. IBM SPSS software (v26.0) was used for statistical analyses.

## 3 RESULTS

### 3.1 Demographic and clinical characteristics differences between groups

The demographic and clinical characteristics of the diagnosed iNPH and HC groups are summarized in Table 1. There was no significant intergroup difference in age or frame‐wise displacement (FD) distribution (*p*1 = 0.612; *p*2 = 0.439). As there was a significant difference (*p* = 0.003) in gender distribution, we included gender as a covariable in all statistical analyses. Poor performance was found in patients with iNPH in the Mini‐Mental State Examination (MMSE), Time Up and Go Test (TUG) and 3 subitems of iNPH grading scale (iNPHGS), that is, cognition (iNPHGS_c), motion (iNPHGS_m), and urination (iNPHGS_u) (*p* < 0.001).

**TABLE 1:** Demographic and clinical data of iNPH patients and participants.

Characteristics	iNPH (*n* = 33)		HC (*n* = 23)	*p*1 value (pre‐operation vs. HC)	*p*2 value (pre‐operation vs. post‐)
	Preoperative	3 months postoperative			
Age (average, range)	74.45 ± 8.78		75.57 ± 6.73	0.612	NS
Gender (male/female)	26/7		9/14	0.003**	NS
FD	0.190 (0.103)		0.196 (0.132)	0.439	NS
INPHGS					
Motion	2 (0)	2 (1)	0 (0.25)	<0.001**	<0.001**
Cognition	2 (0)	2 (0.75)	0 (1)	<0.001**	<0.001**
Urination	2 (1)	1 (1)	0 (0)	<0.001**	<0.001**
Total	7.12 ± 2.60	4.52 ± 2.11	0.57 (0.59)	<0.001**	<0.001**
MMSE	14.91 ± 9.41	17.24 ± 9.16	28.96 (1.26)	<0.001**	<0.001**
TUG‐t	27.84 (19.58)	18.94 (15.18)	9.79 (1.69)	<0.001**	<0.001**

*Note*: Data are presented as mean ± SD or median (quartile); **p < 0.001.

Abbreviations: FD, frame‐wise displacement; HC, healthy control; iNPH, idiopathic normal pressure hydrocephalus; INPHGS, idiopathic normal pressure hydrocephalus grading scale; MMSE, Mini‐Mental State Examination; ns, no significance; TUG‐t, timed up and go test.

### 3.2 Intrinsic connectivity networks

**FIGURE 1:**
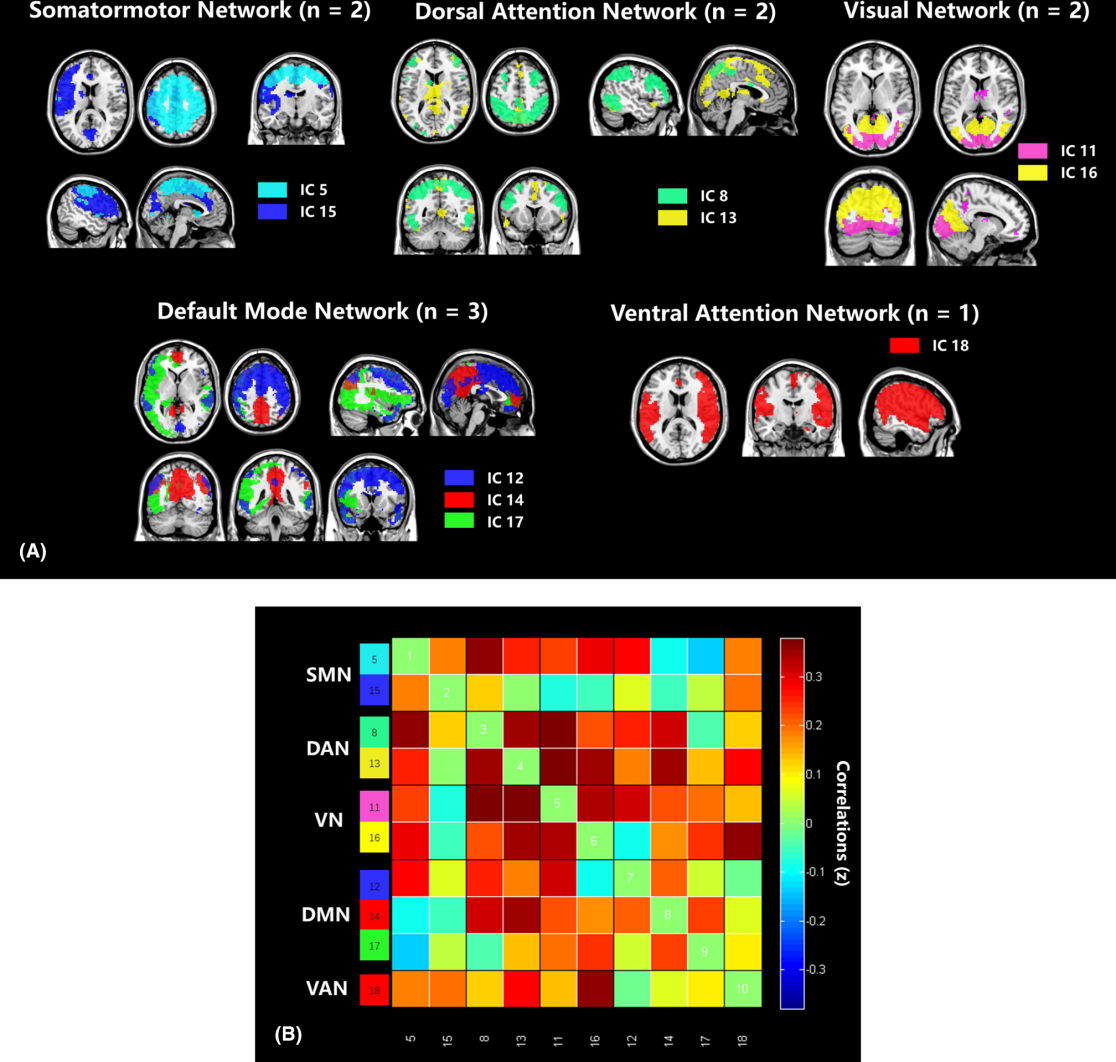
(A) Independent components (*n* = 10) identified by group independent component analysis. Independent component special maps divided on seven functional networks [somatormotor network (SMN), dorsal attention network (DAN), visual network (VN), default mode network (DMN), ventral attention network (VAN)] based on their anatomical and functional properties. (B) Group averaged static FC between IC pairs was computed using the entire resting state data. The value in the correlation matrix represents the Fisher's z‐transformed Pearson correlation coefficient. Each of the 10 ICs was rearranged by network group based on the seven functional networks. Color‐coded legends of ICs were pasted on the left side of the matrix, which matches to the overlaid colors of the spatial maps in figure A.

Spatial maps of all 10 ICs defined using the group ICA are shown in Figure 1A. ICs were grouped into the following five networks: somatomotor network (SMN; IC5 and 15), dorsal attention network (DAN; IC8 and 13), visual network (VN; IC11 and 16), default mode network (DMN; IC12, 14 and 17), ventral attention network (VAN; IC18). Figure 1A,B display the group averaged static functional connectivity network between ICs computed over the entire scan. The detailed information of independent components was listed in Table S1 in Supplementary Materials.

### 3.3 Group‐discriminating sFNC

**FIGURE 2:**
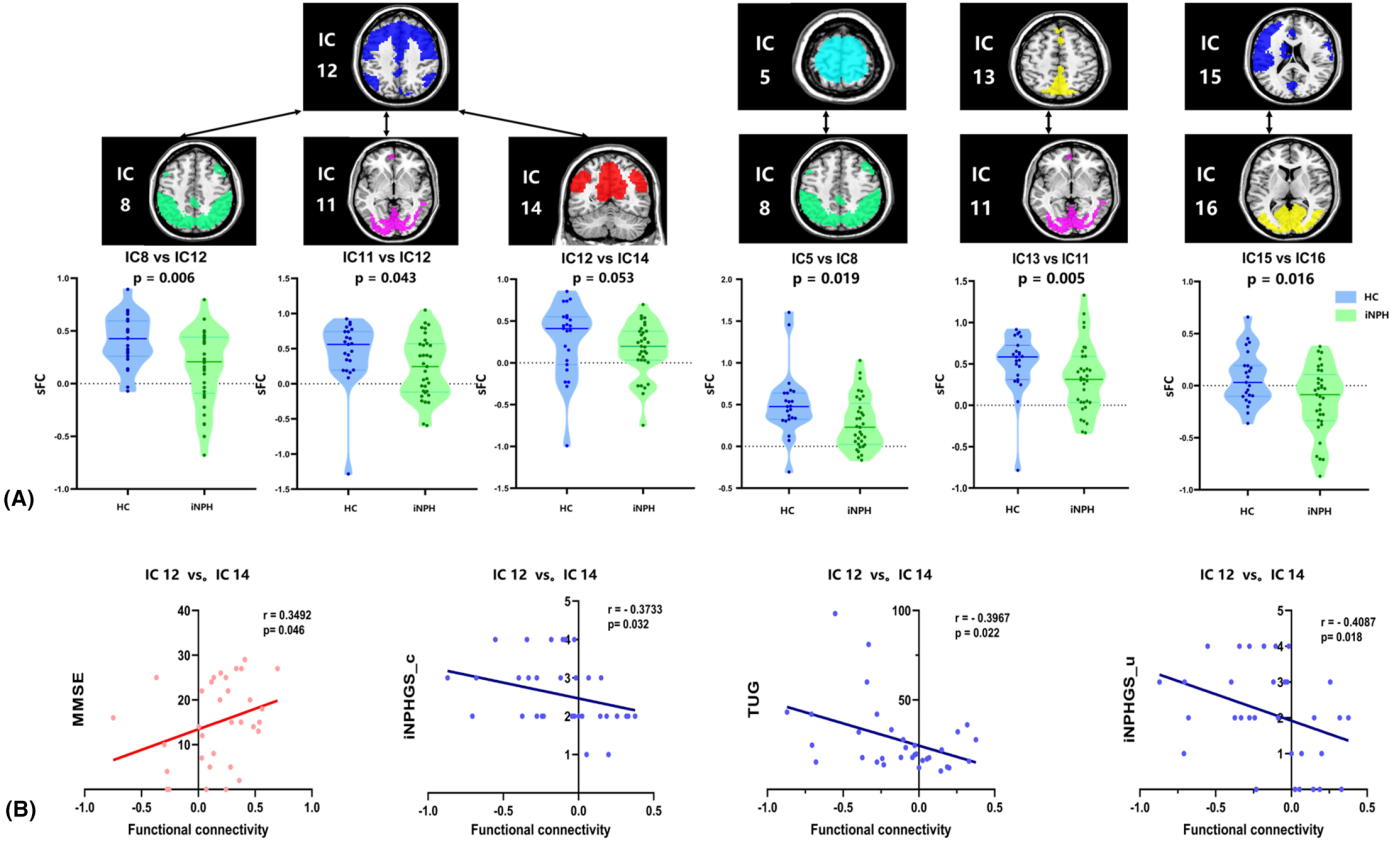
Static functional network connectivity (sFNC) analysis in independent components (ICs). (A) SFNC significantly decreased in iNPH group than HC group among partially ICs after false discovery rate (FDR) correction. (B) Correlation analysis between sFNC among ICs and pre‐ and post‐operational clinical symptoms in iNPH group.

**FIGURE 3:**
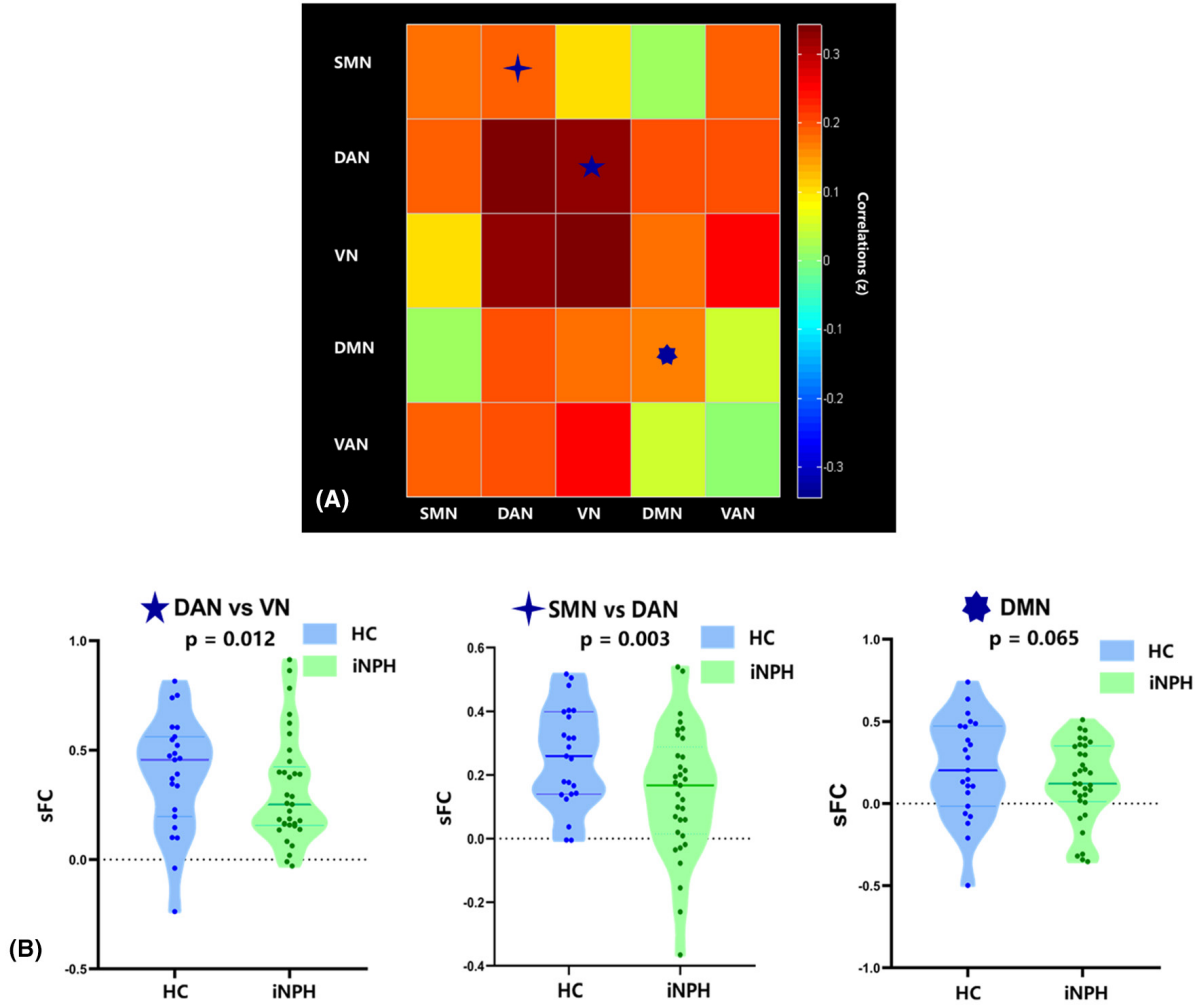
(A) Static functional network connectivity (sFNC) analysis in large‐scale networks. (B) SFNC significantly decreased in iNPH group than HC group among partially large‐scale functional networks, between DAN and VN (five‐pointed star), SMN and DAN (four‐pointed star), and within DMN (seven‐pointed star).

The sFNC between all ICs and large‐scale functional networks is presented as matrices in Figures 1B and 3A. Pairwise comparisons were conducted between each iNPH group and each HC group. Compared with HCs, iNPH participants had significantly decreased sFNC in IC12 versus (vs.) IC8 (*p* = 0.006), IC12 vs.IC11 (*p* = 0.043), IC12 vs. IC14 (*p* = 0.053), IC5 vs. IC8 (*p* = 0.019), IC13 vs. IC11 (*p* = 0.005) and IC15 vs. IC16 (*p* = 0.016) (Figure 2A). The sFNC also had a significant decrease in iNPH in DAN vs.VN (*p* = 0.012) and SMN vs. DAN (*p* = 0.003) and a borderline significant decrease within SMN (*p* = 0.065) (Figure 3B).

### 3.4 State analysis and functional connectivity strength in the dFNC

**FIGURE 4:**
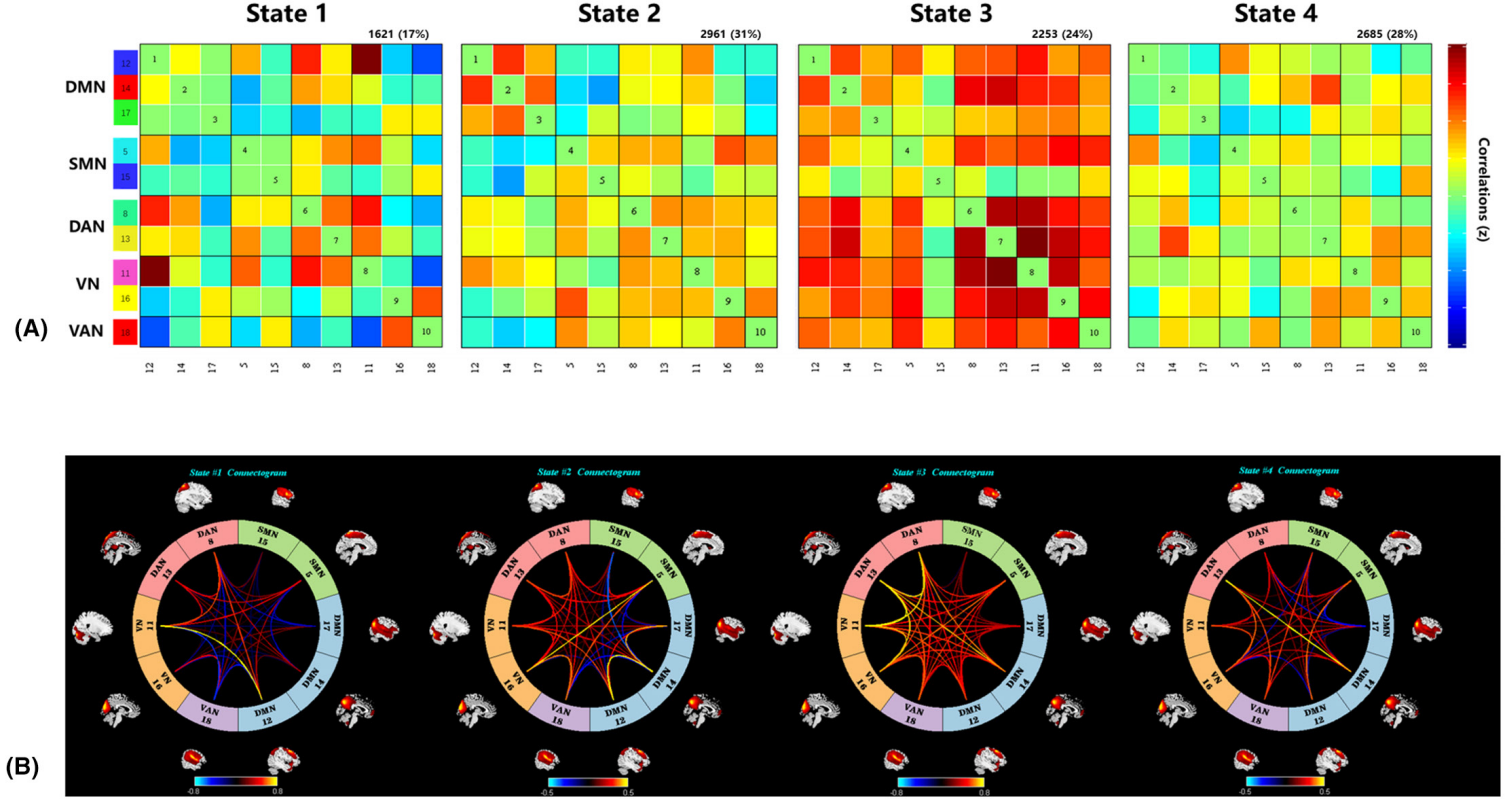
(A) Results of the clustering analysis per state. The total number of occurrences and percentage of total occurrences are listed above each cluster median [percentage of total occurrences for state‐1, state‐2, state‐3, state‐4: 17%, 31%, 24%, and 28%]. (B) The corresponding visualized connectivity patterns of these four FC states.

The centroids and the count of dFNC states are displayed in Figure 4A. The most frequent connected state‐2 (31%), and a least frequent interconnected state‐1 (17%). The percentage of total occurrences of another two states was similar with state‐2, with state‐3 (24%) and state‐4 (28%). Figure 4B showed the corresponding visualized connectivity patterns of these four FC states. In state‐3, connections between ICs were located only within networks with positive correlations, especially between and within DAN and VN. Similarly, the connections between ICs in state‐2 and state‐4 were mainly located within networks with positive couplings, especially connections between IC13 in DAN and IC14 in DMN (state‐4) and within DMN (state‐2). In contrast, the least and sparsely connected state (state‐1) was characterized by connections between functional connectivity networks with positive and negative correlations.

### 3.5 Average instantaneous functional connectivity of ICs in the iNPH and HC groups

**FIGURE 5:**
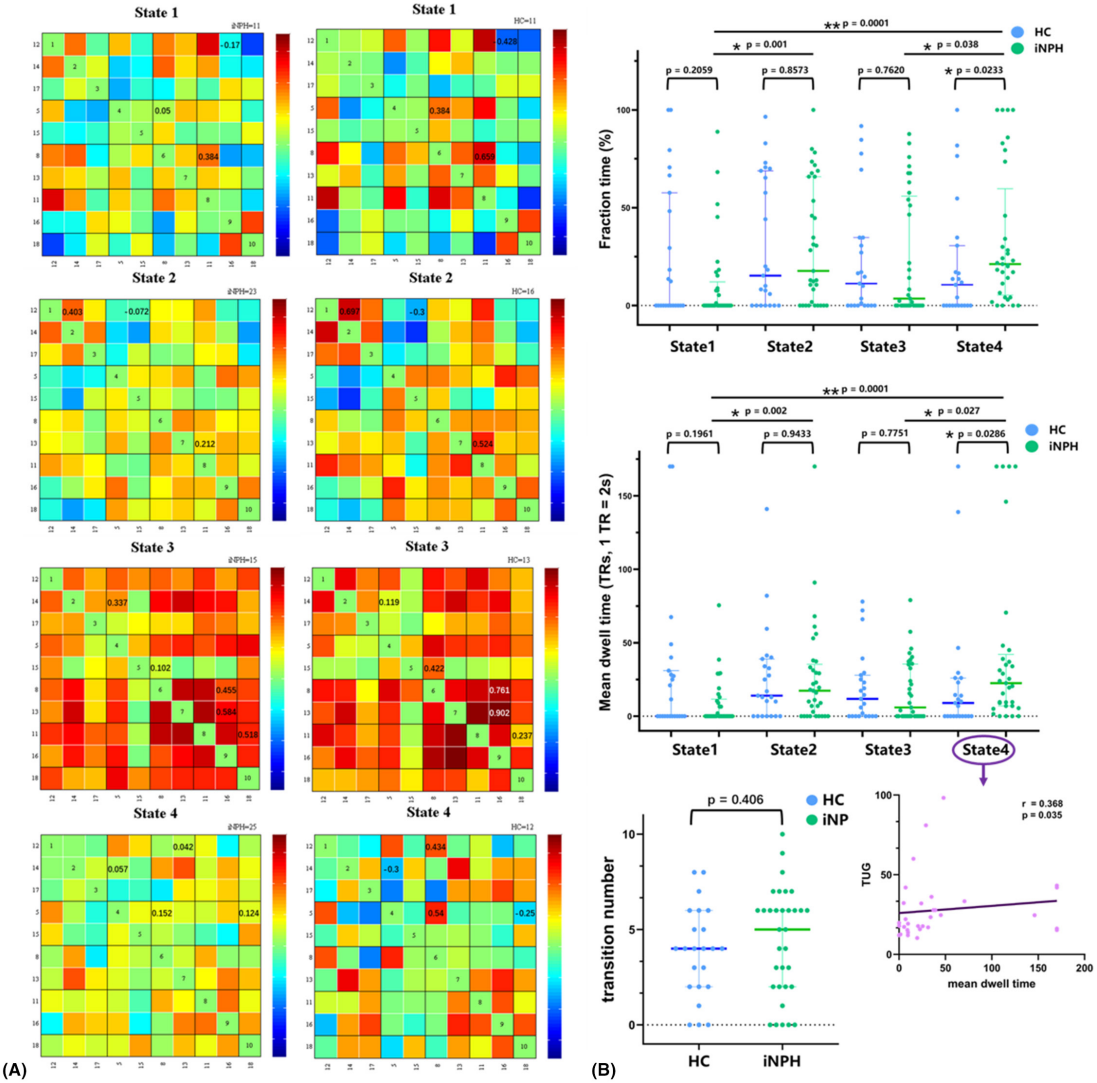
Comparison of the temporal properties of the dynamic FC states between the iNPH and HC groups. (A) The cluster centroid matrices of four dFNC states, along with the count of participants that have at least one window clustered into each state [total occurrences for state‐1, state‐2, state‐3, state‐4: 11, 23, 15, and 25 in the iNPH group and 11, 16, 13, and 12 in the HC group, respectively]; group comparison in average instantaneous FC of ICs between iNPH and HC group, along with the average instantaneous FC values with significant between‐group differences are annotated in the matrixes (*p* < 0.05). (B) Comparison of group level and state level in fraction time, mean dwell time and transition number of four dynamic functional network connectivity (dFNC) states. Asterisks indicating significant group difference. Only the mean dwell time in state‐4 had significant correlation with the TUG score.

Figure 5A showed state‐ and group‐specific cluster centroids. Average instantaneous FCs of ICs were calculated and compared between the iNPH and HC groups, and we observed that compared with HCs, the iNPH group had a significantly decreased average instantaneous FC in most ICs for both positive and negative correlation networks, except for IC18 vs.IC11 (iNPH: 0.518, HC: −0.237, *p* = 0.002) and IC5 vs.IC14 (iNPH: 0.337, HC: 0.119, *p* = 0.009) in state‐3. Detailed information was displayed in Table S2 in Supplementary Materials.

### 3.6 Temporal properties of states in the iNPH and HC groups

We observed that in the iNPH and HC participants, state‐3 was characterized by positive internetwork connections, involving the DAN, VN and DMN. In the iNPH group, state‐4 was most frequently observed (*n* = 25); whereas in HCs, state‐4 occurred less frequently (*n* = 12). State‐1 was the least frequently observed state in both the iNPH (*n* = 11) and HC groups (*n* = 11) (Figure 5A).

Pairwise group comparisons in the fraction time of occurrences and the mean dwell time are shown in Figure 5B. The results indicated a significant group difference in fractional time and mean dwell time of state‐4 (*p* < 0.05, Mann–Whitney *U*‐test; median, 25%–75%), which suggests that iNPH exhibited an increased proportion of time spent only in state‐4 compared to HC. Specifically, among the four states, iNPH had a significantly longer fraction time (iNPH: 21.18, 5.295–59.71; HC: 10.59, 0–30.59; *p* = 0.023) and mean dwell time (iNPH: 22.5, 7–42; HC: 9, 0–26; *p* = 0.029) in state‐4 than in HC. Between‐state comparisons in the iNPH group revealed that iNPH participants spent significantly most time in state‐4 and least time in state‐1 (fraction time: 0, 0–12.06; mean dwell time: 0, 0–11.67).

The distribution of state transition times displayed no difference between iNPH and HC group (*p* = 0.406, Figure 5B).

### 3.7 Analyses on regional temporal variability

**FIGURE 6:**
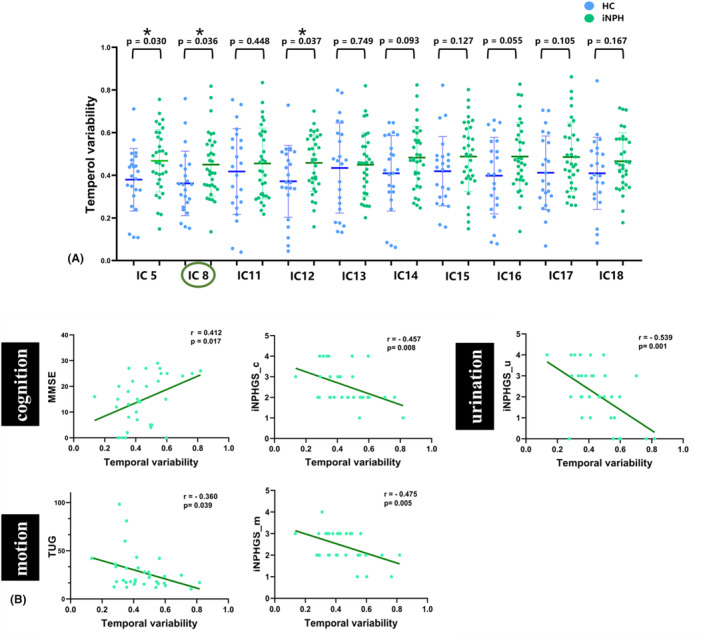
Results of the temporal variability analysis. (A) Group comparison in temporal variability of all ICs. Asterisks indicating significant group difference. (B) Correlation analysis between all group discriminating temporal variability of ICs and preoperational clinical symptoms score of iNPH.

Among the 10 ICs in the brain network, 3 ICs exhibited significantly increased temporal variability in iNPH patients compared HCs (*p* < 0.05, FDR corrected); these ICs comprised IC5 (SMN, *p* = 0.030), IC8 (DAN, *p* = 0.036), and IC12 (DMN, *p* = 0.037). The temporal variability of IC16 in the VN had a borderline significant increase in iNPH (*p* = 0.055; Figure 6A).

### 3.8 Associations of sFNC and dFNC features with the clinical performance of iNPH

Correlation analyses were carried out to test whether sFNC and dFNC properties were associated with clinical characteristics. A marginally significant positive correlation was observed between the preoperative MMSE scores of iNPH patients and the median strength of the significantly decreased sFNC between IC12 vs. IC14 (*r* = 0.349, *p* = 0.046). The preoperative iNPHGS_c, iNPHGS_u, and TUG scores showed a significant negative correlation with sFNC between IC15 vs. IC16, respectively (*r*1 = −0.373, *p*1 = 0.032; *r*2 = −0.397, *p*2 = 0.022; *r*3 = −0.409, *p*3 = 0.018, Figure 2B). After correction for multiple comparisons, there was a no significant association between group‐discriminating sFNC in the large‐scale network and clinical symptoms.

In a further analysis of correlations between dFNC properties, that is, fraction time, mean dwell time and transition number, and clinical characteristics in the iNPH group, we found no significant results after multiple comparison correction expect for the mean dwell time in state‐4 and the TUG score (*r* = 0.368, *p* = −0.035). Regarding temporal variability, the clinical performance of iNPH patients showed a significant correlation with the group discriminating temporal variability of ICs, that is, IC8. Specifically, higher temporal variability in IC8 was associated with worse cognitive performance, including MMSE score (*r* = 0.412, *p* = 0.017) and iNPHGS_c scores (*r* = −0.457, *p* = 0.008; Figure 6B). Moreover, the scores of TUG and iNPHGS_m were negatively correlated with the temporal variability in IC8 (*r*1 = −0.36, *p*1 = 0.039; *r*2 = −0.475, *p*2 = 0.005; Figure 6B). The urination performance, as measured by the iNPHGS_u, also showed a significant negative correlation with regional temporal variability in IC8 (*r* = −0.539, *p* = 0.001). The abovementioned results may indicate an association between the worsening of clinical function and group‐discriminating temporal variability.

## 4 DISCUSSION

The present study employed both sFNC and dFNC analysis to clarify rs‐FC changes in iNPH; another purpose was to determine the association between these changes and clinical presentation. A dynamic analysis framework, based on ICA, Pearson correlation coefficient, sliding window approach and *k*‐means clustering, is a method to analyze the dynamic changes in relative FNC among networks.^13^, ^14^ This is the first study in iNPH cohort to combine a dynamic analysis framework with classical sFNC to investigate changes in brain connectivity from both static and dynamic perspectives. Our results highlight that (1) the sFNC of brain networks is generally decreased in iNPH patients, and the reduction within the DMN and between the SMN and VN is related to clinical symptoms; (2) state‐4 is the iNPH‐abnormality state, and the temporal properties and temporal variability in state‐4 are sensitive to identify iNPH; (3) the dFNC analysis can supplement the sFNC with additional information, and investigate rs‐FC from both static and dynamic perspectives can help to depict a full picture of rs‐FC abnormalities in iNPH.

The sFNC of brain networks in iNPH patients is generally decreased. The dysfunction that occurs in iNPH could stem from distinct changes in the information processing in the brain underlying disparate network damage. Decreased FC within the DMN significantly correlated with cognitive decline, which is consistent with the results of previous studies.^6^, ^23^, ^24^, ^25^ Interestingly, we found that the decrease in internal connectivity of the DMN was more significant than that in FC among other networks. The reduction in FC within the DMN showed weaker significance than it in other networks. A study of iNPH in 2020 found that changes in FC in the DMN detected by FDG‐PET were associated with the course of iNPH, and the decreased FC occurred after the onset of clinical symptoms, which indicated that the disease had entered a decompensated stage.^26^ The abnormality of connectivity intensity within the DMN can help to capture reliable biomarkers of the severity of clinical symptoms and advance the current understanding of the pathological mechanism underlying iNPH. On the other hand, a reduction in connectivity between the SMN and VN was associated with the poor cognitive and motor performance. The disconnection between SMN and VN may be the key to the appearance of symptoms in iNPH.

Dynamic FC may reflect aspects of the capacity of neural system functional^27^ and serve as another physiological biomarker of neurodegenerative disease.^28^, ^29^ The highly variable dFNC patterns depart significantly from those of sFNC, which imply a flexible diversity of functional coordination between subsystems.^30^ The dFNC analyses of the present study suggested four discrete connectivity configurations. The temporal properties of the iNPH and HC groups differed across dFNC states. The results showed that both iNPH and HC group had the shortest dwell times in state‐1 with sparse connections. It highly indicated that FC and network integrity changes in iNPH overlap with normal aging, but the decline was more rapid in iNPH patients. Only high‐strength positive connections were present in state‐3, or the integration state. The reduced number of transitions to the integration state in iNPH is consistent with results obtained in other neurological disorders.^29^, ^31^ This may suggest that the segregation of neural network and functional connection may be closely related to the pathogenesis of iNPH.

Importantly, iNPH patients had the longest dwell time at state‐4, named the iNPH‐abnormality state. The longer dwell time in the iNPH‐abnormality state in iNPH suggested that it may cost more energy to process the externally derived input information and execute the output. Taking the above evidence together, we suggest that the iNPH‐abnormality state may be a core working state specific to iNPH and that an increased frequency of this state would facilitate functional classification. In addition, we observed that iNPH patients transitioned more frequently than HCs did, and the distribution of transition frequencies indicated that iNPH patients were more likely to transition to the iNPH‐abnormality state than HCs, reflecting a propensity for this state. The iNPH patients with longer mean dwell time in the iNPH‐abnormality state presented poorer gait performance, and the cluster result showed the decrease in FC between the DMN and DAN was more significant in the iNPH‐abnormality state than in other states. We speculated that the increased time spent in the iNPH‐abnormality state would affect the exchange of information between the DMN and DAN, and that the decline of cognition (associated with the DMN) and attention (associated with the DAN) may be the cause of motor disturbance in iNPH patients. In addition, the DAN is involved in the formation of the somatormotor system, consistent with the local processing of motor output,^32^ which supports the former conjecture.

INPH patients experienced more state transitions and had higher regional temporal variability than HCs, probably because the FC matrices derived from the iNPH group tended to have higher overall diversity. The increased temporal variabilities in the SMN, DMN and DAN were related to compensation for declines in sensory, motor, and cognitive function, consistent with a previous report that the brain may compensate for cognitive decline by enhancing the variability of corresponding networks.^33^ The temporal variability in IC8 of the DAN was significantly correlated with clinical triad symptoms in iNPH patients, implying that the vulnerability of the DAN may be closely related to disease expression. The DAN is sensitive to the effects of aging and is considered to underlie the degradation of attention that occurs with aging.^34^ The accelerated degeneration of iNPH and the clinical correlation of increased temporal variability of the DAN suggested that impaired attention in iNPH may be the core cause of symptom emergence. The instability of interactions between the DAN and other networks may hinder the expression of corresponding cognitive, motor, and urinary functions.

The decrease in instantaneous FC in dFNC was consistent with the intrinsic FC in sFNC. FC reduction was demonstrated from both static and dynamic perspectives, not only among networks but also within the DMN. This may be one of the characteristic changes in iNPH, which is related to the damage to the brain networks. Decreased FC of the SMN, DMN, and DAN was found in all four FC states, suggesting that these three networks may be the key to the whole brain affects of iNPH. Decreases in FC in the DAN could underlie the impairments in sustained attention, which is one of the most affected cognitive functions that occurs with aging.^34^ The regulation of activity in the DMN is also critical and closely related to the key interactive functional network that coordinates motor and cognitive functions.^35^ The interaction between the DMN and SMN is impaired in elderly, suggesting that DMN‐SMN co‐deactivation may relate to gait impairments and fall risk in aging.^36^ Attention, sensory and cognition dysfunction interact with each other, further aggravating the clinical symptoms of iNPH, which provides additional support for our previous view.

The increased connectivity between the VAN and VN in iNPH group may be related to compensatory mechanisms. Combined with the results of sFNC, we speculate that the VN is another major sensory domain affected by iNPH. It has been proven that visual–sensory–motor interaction is essential for motion control^37^; thus, visual dysfunction may be one of the causes of gait disorder in iNPH. This result partially supports our previous findings regarding sFNC, that is, decreased FC between the SMN and VN is correlated with poor motion performance. Dynamic FNC may be more sensitive than sFNC to changes in overall FNC, providing us with extra information on the mechanisms underlying the effects of iNPH on networks.

There are a few limitations that should be considered in the interpretation of our results. First, due to the limited sample size, we did not have sufficient statistical power to investigate the association between dFNC characteristics and clinical scores separately. As more scan data are collected in the future, the exact relationship between dFNC features and symptoms can be more accurately demonstrated. Another limitation is the difference in gender distribution between patients and HCs. Although we corrected for the effect of gender by including it as a covariate of no interest in the regression model, residual effects of gender may still exist or have nonlinear effects on functional networks. Finally, future studies could use rs‐fMRI data in different stages of diagnosed iNPH to examine the relationship of functional integration and separation with symptoms, which could contribute to the understanding of the mechanisms of FNC change in iNPH.

## AUTHOR CONTRIBUTIONS

All authors contributed to the final manuscript of this study. Conceptualization, methodology, and formal data analysis were completed by Wenjun Huang. Data collection and investigation were performed by Xuhao Fang. Wenjun Huang wrote the first draft of the manuscript. Review and editing of the manuscript were carried out by all co‐authors. Guangwu Lin was responsible for the overall supervision.

## FUNDING INFORMATION

The National Natural Science Foundation of China (NSFC), Grant/Award Number: 81771816; Hospital Development Centre, Grant/Award Number: SHDC2022DRT025, SKLY2022CRT402; Shanghai municipal population and family planning commission, Grant/Award Number: 202240257 and 201740003; Investigator‐Initiated Research Projects of Huadong Hospital, Grant/Award Number: HDLC2022003.

## CONFLICT OF INTEREST STATEMENT

None of the authors has any conflicts of interest to disclosure.

## Supporting information


Appendix S1

Table S1

Table S2

## Data Availability

Because of the privacy regulations of the hospital, the clinical data and MRI data can only be accessible by contacting Dr. Wenjun Huang with research application.

## References

[1] Hakim S , Adams RD . The special clinical problem of symptomatic hydrocephalus with normal cerebrospinal fluid pressure. Observations on cerebrospinal fluid hydrodynamics. J Neurol Sci. 1965;2:307‐327. 5889177 10.1016/0022-510x(65)90016-x

[2] Nakajima M , Yamada S , Miyajima M , et al. Guidelines for management of idiopathic normal pressure hydrocephalus (third edition): endorsed by the Japanese society of normal pressure hydrocephalus. Neurol Med Chir. 2021;61:63‐97. 10.2176/nmc.st.2020-0292 PMC7905302 33455998

[3] Rosenbaum RB . Normal pressure hydrocephalus: how often does the diagnosis hold water? Neurology. 2012;78:152; author reply 152. 10.1212/01.wnl.0000410914.88642.71 22232055

[4] Junkkari A , Häyrinen A , Rauramaa T , et al. Health‐related quality‐of‐life outcome in patients with idiopathic normal‐pressure hydrocephalus – a 1‐year follow‐up study. Eur J Neurol. 2017;24:58‐66. 27647684 10.1111/ene.13130

[5] Popal AM , Zhu Z , Guo X , et al. Outcomes of Ventriculoperitoneal shunt in patients with idiopathic Normal‐pressure hydrocephalus 2 years after surgery. Front Surgery. 2021;8:641561. 10.3389/fsurg.2021.641561 PMC8634250 34869547

[6] Khoo HM , Kishima H , Tani N , et al. Default mode network connectivity in patients with idiopathic normal pressure hydrocephalus. J Neurosurg. 2016;124:350‐358. 26295919 10.3171/2015.1.JNS141633

[7] Ogata Y , Ozaki A , Ota M , et al. Interhemispheric resting‐state functional connectivity predicts severity of idiopathic Normal pressure hydrocephalus. Front Neurosci. 2017;11:470. 28919849 10.3389/fnins.2017.00470 PMC5585196

[8] Takamura T , Hanakawa T . Clinical utility of resting‐state functional connectivity magnetic resonance imaging for mood and cognitive disorders. J Neural Transm (Vienna, Austria: 1996). 2017;124:821‐839. 10.1007/s00702-017-1710-2 28337552

[9] Bommarito G , Van De Ville D , Frisoni GB , et al. Alzheimer's disease biomarkers in idiopathic Normal pressure hydrocephalus: linking functional connectivity and clinical outcome. J Alzheimer's Dis. 2021;83:1717‐1728. 34459399 10.3233/JAD-210534

[10] Allen EA , Damaraju E , Plis SM , Erhardt EB , Eichele T , Calhoun VD . Tracking whole‐brain connectivity dynamics in the resting state. Cerebral Cortex (New York, NY: 1991). 2014;24:663‐676. 10.1093/cercor/bhs352 PMC3920766 23146964

[11] Fu Z , Caprihan A , Chen J , et al. Altered static and dynamic functional network connectivity in Alzheimer's disease and subcortical ischemic vascular disease: shared and specific brain connectivity abnormalities. Hum Brain Mapp. 2019;40:3203‐3221. 30950567 10.1002/hbm.24591 PMC6865624

[12] Yu Q , Erhardt EB , Sui J , et al. Assessing dynamic brain graphs of time‐varying connectivity in fMRI data: application to healthy controls and patients with schizophrenia. Neuroimage. 2015;107:345‐355. 25514514 10.1016/j.neuroimage.2014.12.020 PMC4300250

[13] Fu Z , Tu Y , Di X , et al. Characterizing dynamic amplitude of low‐frequency fluctuation and its relationship with dynamic functional connectivity: an application to schizophrenia. Neuroimage. 2018;180:619‐631. 28939432 10.1016/j.neuroimage.2017.09.035 PMC5860934

[14] Fu Z , Tu Y , Di X , et al. Transient increased thalamic‐sensory connectivity and decreased whole‐brain dynamism in autism. Neuroimage. 2019;190:191‐204. 29883735 10.1016/j.neuroimage.2018.06.003 PMC6281849

[15] Kim J , Criaud M , Cho SS , et al. Abnormal intrinsic brain functional network dynamics in Parkinson's disease. Brain: A J Neurol. 2017;140:2955‐2967. 10.1093/brain/awx233 PMC5841202 29053835

[16] Du Y , Fryer SL , Fu Z , et al. Dynamic functional connectivity impairments in early schizophrenia and clinical high‐risk for psychosis. Neuroimage. 2018;180:632‐645. 29038030 10.1016/j.neuroimage.2017.10.022 PMC5899692

[17] Yeo BT , Krienen FM , Sepulcre J , et al. The organization of the human cerebral cortex estimated by intrinsic functional connectivity. J Neurophysiol. 2011;106:1125‐1165. 21653723 10.1152/jn.00338.2011 PMC3174820

[18] Shirer WR , Ryali S , Rykhlevskaia E , Menon V , Greicius MD . Decoding subject‐driven cognitive states with whole‐brain connectivity patterns. Cerebral Cortex (New York, NY: 1991). 2012;22:158‐165. 10.1093/cercor/bhr099 PMC3236795 21616982

[19] Smith SM , Miller KL , Salimi‐Khorshidi G , et al. Network modelling methods for FMRI. Neuroimage. 2011;54:875‐891. 20817103 10.1016/j.neuroimage.2010.08.063

[20] Friedman J , Hastie T , Tibshirani R . Sparse inverse covariance estimation with the graphical lasso. Biostatistics (Oxford, England). 2008;9:432‐441. 18079126 10.1093/biostatistics/kxm045 PMC3019769

[21] Zhang J , Cheng W , Liu Z , et al. Neural, electrophysiological and anatomical basis of brain‐network variability and its characteristic changes in mental disorders. Brain: A J Neurol. 2016;139:2307‐2321. 10.1093/brain/aww143 27421791

[22] Gu Y , Lin Y , Huang L , et al. Abnormal dynamic functional connectivity in Alzheimer's disease. CNS Neurosci Ther. 2020;26:962‐971. 32378335 10.1111/cns.13387 PMC7415210

[23] Whitfield‐Gabrieli S , Ford JM . Default mode network activity and connectivity in psychopathology. Annu Rev Clin Psychol. 2012;8:49‐76. 22224834 10.1146/annurev-clinpsy-032511-143049

[24] Badhwar A , Tam A , Dansereau C , Orban P , Hoffstaedter F , Bellec P . Resting‐state network dysfunction in Alzheimer's disease: a systematic review and meta‐analysis. Alzheimer's Dementia (Amsterdam, Netherlands). 2017;8:73‐85. 10.1016/j.dadm.2017.03.007 PMC5436069 28560308

[25] Grieder M , Wang DJJ , Dierks T , Wahlund LO , Jann K . Default mode network complexity and cognitive decline in mild Alzheimer's disease. Front Neurosci. 2018;12:770. 30405347 10.3389/fnins.2018.00770 PMC6206840

[26] Griffa A , Bommarito G , Assal F , Herrmann FR , Van De Ville D , Allali G . Dynamic functional networks in idiopathic normal pressure hydrocephalus: alterations and reversibility by CSF tap test. Hum Brain Mapp. 2021;42:1485‐1502. 33296129 10.1002/hbm.25308 PMC7927299

[27] Kucyi A , Hove MJ , Esterman M , Hutchison RM , Valera EM . Dynamic brain network correlates of spontaneous fluctuations in attention. Cerebral Cortex (New York, NY: 1991). 2017;27:1831‐1840. 10.1093/cercor/bhw029 PMC6317462 26874182

[28] Cordes D , Zhuang X , Kaleem M , et al. Advances in functional magnetic resonance imaging data analysis methods using empirical mode decomposition to investigate temporal changes in early Parkinson's disease. Alzheimer's Dementia (New York, N Y). 2018;4:372‐386. 10.1016/j.trci.2018.04.009 PMC6115608 30175232

[29] Díez‐Cirarda M , Strafella AP , Kim J , et al. Dynamic functional connectivity in Parkinson's disease patients with mild cognitive impairment and normal cognition. NeuroImage Clin. 2018;17:847‐855. 29527489 10.1016/j.nicl.2017.12.013 PMC5842729

[30] Marusak HA , Calhoun VD , Brown S , et al. Dynamic functional connectivity of neurocognitive networks in children. Hum Brain Mapp. 2017;38:97‐108. 27534733 10.1002/hbm.23346 PMC5796541

[31] Liu F , Wang Y , Li M , et al. Dynamic functional network connectivity in idiopathic generalized epilepsy with generalized tonic‐clonic seizure. Hum Brain Mapp. 2017;38:957‐973. 27726245 10.1002/hbm.23430 PMC6866949

[32] Russo D , Martino M , Magioncalda P , Inglese M , Amore M , Northoff G . Opposing changes in the functional architecture of large‐scale networks in bipolar mania and depression. Schizophr Bull. 2020;46:971‐980. 32047938 10.1093/schbul/sbaa004 PMC7342167

[33] Zhao C , Huang WJ , Feng F , et al. Abnormal characterization of dynamic functional connectivity in Alzheimer's disease. Neural Regen Res. 2022;17:2014‐2021. 35142691 10.4103/1673-5374.332161 PMC8848607

[34] Tomasi D , Volkow ND . Aging and functional brain networks. Mol Psychiatry. 2012;17(471):549‐458. 10.1038/mp.2011.81 PMC3193908 21727896

[35] Fox MD , Raichle ME . Spontaneous fluctuations in brain activity observed with functional magnetic resonance imaging. Nat Rev Neurosci. 2007;8:700‐711. 17704812 10.1038/nrn2201

[36] Rodriguez‐Sabate C , Morales I , Sanchez A , Rodriguez M . The functional interaction of the brain default network with motor networks is modified by aging. Behav Brain Res. 2019;372:112048. 31288062 10.1016/j.bbr.2019.112048

[37] Glickstein M . How are visual areas of the brain connected to motor areas for the sensory guidance of movement? Trends Neurosci. 2000;23:613‐617. 11137151 10.1016/s0166-2236(00)01681-7

