# Supplementary material for: Abnormal characteristic static and dynamic functional network connectivity in idiopathic normal pressure hydrocephalus
Source: CNS Neurosci Ther. 2023 Mar 22;30(3):e14178. doi: 10.1111/cns.14178 (PMC10915979; doi:10.1111/cns.14178)
Supplement: Supplementary file 1 — Appendix S1 [file CNS-30-e14178-s002.docx]

**Supplementary material**

**Inclusion and exclusion criteria and results for participants**

The inclusion criteria in this study were as follows: 1) > 60 years of age; 2) presentation of at least 1 of the following: gait disturbance, dementia, urinary incontinence; 3) ventricular enlargement (Evans Index > 0.3) with narrowing of the subarachnoid space over the high-convexity (DESH); 4) CSF pressure < 200 mmH2O and normal CSF content; 5) improvement of symptoms after CSF-TT and shunt surgery. Exclusion criteria were: 1) cerebral infarction and dementia caused by clear causes and hospitalization for severe mental illness; 2) orthopedic disorders interfering with gait; 3) preceding diseases possibly causing ventricular dilation (including subarachnoid hemorrhage, meningitis, head injury, congenital/developmental hydrocephalus, and aqueductal stenosis); 4) a diagnosis of secondary normal pressure hydrocephalus. On the basis of these criteria, 14 patients were excluded owing to head injury, acute stroke and extensive motion artifacts. Thus, 33 patients with diagnosed iNPH (26 male,7 female; 74.03 ± 8.94) were included in this study. Twenty-three elderly healthy control subjects (14 male,9 female; 75.57 ± 6.66) were included for comparison. The two groups had no significant demographic differences in age (p > 0.05).

**Clinical symptoms assessments**

For all iNPH patients, clinical symptoms were examined by two trained neurologists prior to performing CSF-TT and 3 months after shunt surgery. Besides a routine neurologic examination, the cognitive function was tested with the Mini-Mental State Examination (MMSE),^1^ motor function with a Time Up and Go Test (TUG)^2^ in which time were measured. The overall severity of iNPH was adequately evaluated by the used iNPH grading scale (iNPHGS)^3^ as a subjective evaluation scale. The overall severity of each symptom was rated using the cognition, motion, and urination domains of the iNPHGS, each of the iNPH-specific clinical symptoms (iNPHGS_c, iNPHGS_m, iNPHGS_u) evaluated on a scale from 0 (none) to 4 (severe). The total score (iNPHGS) can be used as an index, together with the evaluation points for each of the three conditions.

The clinical status of all the HCs were also evaluated according to the above protocol.

**MR Imaging Acquisition**

MR imaging data were obtained with a 3-T whole-body MRI scanner (MAGNETOM Prisma, Siemens Healthcare, Erlangen, Germany) by using a 32-channel head coil. Thirty-three patients and 23 healthy controls were scanned in 2021-2022 with the rs-fMRI data as following: repetition time msec/echo time msec, 2000/30; flip angle, 80°; field of view, 192 × 192; slice thickness, 2.0 mm with 0.5 mm gap; 64 slices; spatial resolution, 2.04 mm × 2.04 mm × 2 mm with 205 continuous EPI functional volume scan and an acquisition time of 7 minutes 3 seconds.

For coregistration and normalization of resting-state functional MR imaging data, three-dimensional T1-weighted magnetization-prepared rapid gradient-echo imaging (repetition time msec/echo time msec/inversion time msec, 1800/2.37/900; flip angle, 8°; field of view, 250 × 250; spatial resolution, 0.87mm × 0.85mm × 0.85 mm; slice thickness, 0.85 mm with no gap; 208 slices) was performed.

Fitted but comfortable foam padding was used to minimize head motion. During the rs-fMRI scan, the subjects were instructed to relax with their eyes closed, stay awake, and remain still — no specific task was performed in these conditions.

**Functional data were preprocessed using the Data Processing**

Assistant of the Resting-State Functional MR Imaging toolkit Resting-State fMRI Data Analysis Toolkit plus v1.25 (RESTplus v1.25, http://restfmri.net/forum/REST) based on Statistical Parametric Mapping software (SPM12, http://www.fil.ion.ucl.ac.uk/spm) running in MATLAB 2013b (MathWorks, Natick, MA). All the data were processed with the following steps: (1) conversion of data format; (2) discarding of the first 5 volumes to exclude the influence of unstable longitudinal magnetization; (3) correcting the time layer to align the scanning time of all slices to the reference slice; (4) excluding subjects whose head movement exceeds 2.5 mm or rotates more than 2.5° and reorient (no subjects were excluded in this step); (5) all resulting images were spatially normalized to standard Montreal Neurological Institute space using diffeomorphic anatomical registration through exponentiated Lie algebra (DARTEL) implemented in SPM12; (6) removing covariates (24 head movement parameters, white matter signals, and cerebrospinal fluid signals); and (7) spatial smoothing based on the unified segmentation of structural images (Gaussian kernel, full width at half-maximum = 6 mm).

**Group independent component analysis**

First, the data reduction was conducted to decrease computational complexity using a two-stage principal component analysis. Precisely, the preprocessed fMRI data for each subject was first dimension-reduced temporally and then the reduced data from all subjects were concatenated into a single dataset, or the grouped data, along the temporal dimension and passed through another dimension reduction. Second, ICA was performed to decompose the grouped data into automatically estimated 18 independent components (ICs) using an Infomax algorithm.^4^ In this step, the spatial map and the time course of BOLD signal were generated for each IC. This step was repeated 100 times using the ICASSO algorithm for assessing the repeatability or stability of ICs.^5^ Finally, the resulting components were clustered to estimate their reliability and components with values >0.80 were selected. Subject-specific spatial maps and time courses were obtained using the back-reconstruction approach (GICA) and were converted into z-scores.^6^ Among the 18 ICs, we identified relevant intrinsic connectivity networks by using the automatic identification method and the visual screening based on the criteria by Allen et al.^7^: (i) peak coordinates of spatial maps located primarily in grey matter; (ii) no spatial overlap with vascular, ventricular, or susceptibility artefacts; (iii) time courses dominated by low frequency signals (ratio of powers below 0.1 Hz to 0.15 ― 0.25 Hz in spectrum).

| **Supplementary Table 1: Peak activation information of 10 independent components** | | | | | |
| --- | --- | --- | --- | --- | --- |
| **Intrinsic connectivity networks** | | **n** | **MNI coordinate** | | |
|  |  |  | **x** | **y** | **z** |
| **Somatomotor Network (SMN)** | | | | | |
| IC 5 | Parietal lobe, Postcentral gyrus (B) | 8011 | -5.5 | -51.5 | 71.5 |
| IC15 | Middle, inferior and medial frontal gyri (L) | 6738 | -59.5 | -2.5 | 20.5 |
| **Dorsal Attention Network (DAN)** | | | | | |
| IC 8 | Middle frontal gyrus (B), precuneus (B), Inferior parietal lobule (R) | 7818 | -35.5 | -62.5 | 56.5 |
| IC 13 | Fusiform gyrus (B), thalamus (B), precuneus (B) | 3814 | 3.5 | -80 | 47.5 |
| **Visual Network (VN)** | | | | | |
| IC 11 | Lingual gyrus (B), middle occipital gyrus (B) | 3846 | 23.5 | -86.5 | -20.5 |
| IC 16 | Parietal lobe (B), cuneus (B), precuneus (B) | 6541 | 3.5 | -86.5 | 26.5 |
| **Default Mode Network (DMN)** | | | | | |
| IC 12 | Middle and superior frontal gyri (B) | 14488 | -47.5 | 11.5 | 47.5 |
| IC 14 | Precuneus, medial of superior frontal gyrus (B) | 6242 | 2.5 | -71.5 | 41.5 |
| IC 17 | Middle and superior temporal gyri (B), middle occipital gyrus (L), middle and inferior frontal gyri (L) | 10414 | -51.5 | -62.5 | 27.5 |
| **Ventral Attention Network (VAN)** | | | | | |
| IC 18 | Superior temporal gyrus (R), middle and inferior frontal gyri (R), pre- and postcentral gyri (R). | 9887 | 60.5 | -20.5 | 18.5 |

The coordinates are peak voxel coordinates of the one-sample t-test results for each independent component spatial maps of all subject.

IC, independent component; R, right; L, left; M, medial; B, bilateral; n = the cluster size; R, right; L, left; B, bilateral.

| **Supplementary Table 2:** **Average instantaneous functional connectivity of ICs with significance between iNPH and HC group** | | | | | |
| --- | --- | --- | --- | --- | --- |
| **State** | **IC** | **iNPH** | **HC** | **P value** | **T value** |
| **State-1** | IC12 vs. IC16 | -0.166 | -0.428 | 0.044 | -2.147 |
|  | IC 5 vs. IC 8 | 0.05 | 0.384 | 0.006 | 3.09 |
|  | IC 8 vs. IC11 | 0.384 | 0.658 | 0.040 | 2.198 |
| **State-2** | IC12 vs. IC14 | 0.403 | 0.697 | 0.005 | 2.979 |
|  | IC12 vs. IC15 | -0.072 | -0.3 | 0.028 | -2.289 |
|  | IC11 vs. IC13 | 0.212 | 0.524 | 0.006 | 2.896 |
| **State-3** | IC 8 vs. IC16 | 0.455 | 0.761 | 0.005 | 3.042 |
|  | IC11 vs. IC18 | 0.518 | 0.237 | 0.002 | -3.449 |
|  | IC 5 vs. IC14 | 0.337 | 0.119 | 0.009 | -2.807 |
|  | IC 8 vs. IC15 | 0.102 | 0.422 | 0.049 | 2.063 |
|  | IC13 vs. IC16 | 0.584 | 0.902 | 0.003 | 3.254 |
| **State-4** | IC12 vs. IC 8 | 0.041 | 0.434 | 0.013 | 2.604 |
|  | IC 5 vs. IC14 | 0.057 | -0.298 | 0.015 | -2.561 |
|  | IC 5 vs. IC 8 | 0.152 | 0.540 | 0.042 | 2.11 |
|  | IC 5 vs. IC18 | 0.124 | -0.252 | 0.024 | -2.361 |

IC, independent components; iNPH, idiopathic normal pressure hydrocephalus; HC, healthy control.

**REFERENCE**

1. Folstein MF, Folstein SE, McHugh PR. "Mini-mental state". A practical method for grading the cognitive state of patients for the clinician. *Journal of psychiatric research*. Nov 1975;12(3):189-98.

2. Gallagher R, Marquez J, Osmotherly P. Clinimetric Properties and Minimal Clinically Important Differences for a Battery of Gait, Balance, and Cognitive Examinations for the Tap Test in Idiopathic Normal Pressure Hydrocephalus. *Neurosurgery*. Jun 1 2019;84(6):E378-e384.

3. Kubo Y, Kazui H, Yoshida T, et al. Validation of grading scale for evaluating symptoms of idiopathic normal-pressure hydrocephalus. *Dementia and geriatric cognitive disorders*. 2008;25(1):37-45.

4. Bell AJ, Sejnowski TJ. An information-maximization approach to blind separation and blind deconvolution. *Neural computation*. Nov 1995;7(6):1129-59.

5. Himberg J, Hyvärinen A, Esposito F. Validating the independent components of neuroimaging time series via clustering and visualization. *NeuroImage*. Jul 2004;22(3):1214-22.

6. Calhoun VD, Adali T, Pearlson GD, Pekar JJ. A method for making group inferences from functional MRI data using independent component analysis. *Human brain mapping*. Nov 2001;14(3):140-51.

7. Allen EA, Damaraju E, Plis SM, Erhardt EB, Eichele T, Calhoun VD. Tracking whole-brain connectivity dynamics in the resting state. *Cerebral cortex (New York, NY : 1991)*. Mar 2014;24(3):663-76.
