# Supplementary material for: Abnormal characteristic static and dynamic functional network connectivity in idiopathic normal pressure hydrocephalus
Source: CNS Neurosci Ther. 2023 Mar 22;30(3):e14178. doi: 10.1111/cns.14178 (PMC10915979; doi:10.1111/cns.14178)
Supplement: Supplementary file 2 — Table S1 [file CNS-30-e14178-s001.docx]

| **Supplementary Table 1: Peak activation information of 10 independent components** | | | | | |
| --- | --- | --- | --- | --- | --- |
| **Intrinsic connectivity networks** | | **n** | **MNI coordinate** | | |
|  |  |  | **x** | **y** | **z** |
| **Somatomotor Network (SMN)** | | | | | |
| IC 5 | Parietal lobe, Postcentral gyrus (B) | 8011 | -5.5 | -51.5 | 71.5 |
| IC15 | Middle, inferior and medial frontal gyri (L) | 6738 | -59.5 | -2.5 | 20.5 |
| **Dorsal Attention Network (DAN)** | | | | | |
| IC 8 | Middle frontal gyrus (B), precuneus (B), Inferior parietal lobule (R) | 7818 | -35.5 | -62.5 | 56.5 |
| IC 13 | Fusiform gyrus (B), thalamus (B), precuneus (B) | 3814 | 3.5 | -80 | 47.5 |
| **Visual Network (VN)** | | | | | |
| IC 11 | Lingual gyrus (B), middle occipital gyrus (B) | 3846 | 23.5 | -86.5 | -20.5 |
| IC 16 | Parietal lobe (B), cuneus (B), precuneus (B) | 6541 | 3.5 | -86.5 | 26.5 |
| **Default Mode Network (DMN)** | | | | | |
| IC 12 | Middle and superior frontal gyri (B) | 14488 | -47.5 | 11.5 | 47.5 |
| IC 14 | Precuneus, medial of superior frontal gyrus (B) | 6242 | 2.5 | -71.5 | 41.5 |
| IC 17 | Middle and superior temporal gyri (B), middle occipital gyrus (L), middle and inferior frontal gyri (L) | 10414 | -51.5 | -62.5 | 27.5 |
| **Ventral Attention Network (VAN)** | | | | | |
| IC 18 | Superior temporal gyrus (R), middle and inferior frontal gyri (R), pre- and postcentral gyri (R). | 9887 | 60.5 | -20.5 | 18.5 |

The coordinates are peak voxel coordinates of the one-sample t-test results for each independent component spatial maps of all subject.

IC, independent component; R, right; L, left; M, medial; B, bilateral; n = the cluster size; R, right; L, left; B, bilateral.
