# Supplementary material for: Abnormal characteristic static and dynamic functional network connectivity in idiopathic normal pressure hydrocephalus
Source: CNS Neurosci Ther. 2023 Mar 22;30(3):e14178. doi: 10.1111/cns.14178 (PMC10915979; doi:10.1111/cns.14178)
Supplement: Supplementary file 3 — Table S2 [file CNS-30-e14178-s003.docx]

| **Supplementary Table 2:** **Average instantaneous functional connectivity of ICs with significance between iNPH and HC group** | | | | | |
| --- | --- | --- | --- | --- | --- |
| **State** | **IC** | **iNPH** | **HC** | **P value** | **T value** |
| **State-1** | IC12 vs. IC16 | -0.166 | -0.428 | 0.044 | -2.147 |
|  | IC 5 vs. IC 8 | 0.05 | 0.384 | 0.006 | 3.09 |
|  | IC 8 vs. IC11 | 0.384 | 0.658 | 0.040 | 2.198 |
| **State-2** | IC12 vs. IC14 | 0.403 | 0.697 | 0.005 | 2.979 |
|  | IC12 vs. IC15 | -0.072 | -0.3 | 0.028 | -2.289 |
|  | IC11 vs. IC13 | 0.212 | 0.524 | 0.006 | 2.896 |
| **State-3** | IC 8 vs. IC16 | 0.455 | 0.761 | 0.005 | 3.042 |
|  | IC11 vs. IC18 | 0.518 | 0.237 | 0.002 | -3.449 |
|  | IC 5 vs. IC14 | 0.337 | 0.119 | 0.009 | -2.807 |
|  | IC 8 vs. IC15 | 0.102 | 0.422 | 0.049 | 2.063 |
|  | IC13 vs. IC16 | 0.584 | 0.902 | 0.003 | 3.254 |
| **State-4** | IC12 vs. IC 8 | 0.041 | 0.434 | 0.013 | 2.604 |
|  | IC 5 vs. IC14 | 0.057 | -0.298 | 0.015 | -2.561 |
|  | IC 5 vs. IC 8 | 0.152 | 0.540 | 0.042 | 2.11 |
|  | IC 5 vs. IC18 | 0.124 | -0.252 | 0.024 | -2.361 |

IC, independent components; iNPH, idiopathic normal pressure hydrocephalus; HC, healthy control.
